# Ageing transcriptome meta-analysis reveals similarities and differences between key mammalian tissues

**DOI:** 10.18632/aging.202648

**Published:** 2021-02-11

**Authors:** Daniel Palmer, Fabio Fabris, Aoife Doherty, Alex A. Freitas, João Pedro de Magalhães

**Affiliations:** 1 Integrative Genomics of Ageing Group, Institute of Ageing and Chronic Disease, University of Liverpool, Liverpool, UK; 2 School of Computing, University of Kent, Canterbury, Kent, UK; 3 Rostock University Medical Center, Institute for Biostatistics and Informatics in Medicine and Ageing Research, Rostock, Germany

**Keywords:** Artificial Intelligence, functional genomics, machine learning, microarray, mitochondria

## Abstract

By combining transcriptomic data with other data sources, inferences can be made about functional changes during ageing. Thus, we conducted a meta-analysis on 127 publicly available microarray and RNA-Seq datasets from mice, rats and humans, identifying a transcriptomic signature of ageing across species and tissues. Analyses on subsets of these datasets produced transcriptomic signatures of ageing for brain, heart and muscle. We then applied enrichment analysis and machine learning to functionally describe these signatures, revealing overexpression of immune and stress response genes and underexpression of metabolic and developmental genes. Further analyses revealed little overlap between genes differentially expressed with age in different tissues, despite ageing differentially expressed genes typically being widely expressed across tissues. Additionally we show that the ageing gene expression signatures (particularly the overexpressed signatures) of the whole meta-analysis, brain and muscle tend to include genes that are central in protein-protein interaction networks. We also show that genes underexpressed with age in the brain are highly central in a co-expression network, suggesting that underexpression of these genes may have broad phenotypic consequences. In sum, we show numerous functional similarities between the ageing transcriptomes of these important tissues, along with unique network properties of genes differentially expressed with age in both a protein-protein interaction and co-expression networks.

## INTRODUCTION

Knowledge of expression patterns in ageing organisms can be employed as biomarker panels that estimate a ‘transcriptomic age’ [1], in addition to giving insight into the basic processes associated with ageing [2] and serving as a starting point from which to identify drugs and other interventions that may assist with healthy ageing [3].

Comparative analysis of gene expression data across species is a powerful method to determine an expression signature of ageing. Previously meta-analysis of gene expression with age in mammals has identified changes in stress responses, metabolism and immune response genes [4] while meta-analysis of the dietary restriction expression signature has identified novel changes in retinol metabolism and copper-ion detoxification in this ageing modulating process [5].

Here, we have performed a meta-analysis of ageing using the methods of de Magalhães, et al. [4] on 127 microarray and RNA-Seq datasets from humans, mice and rats, and applied machine learning alongside enrichment methods to analyse the results. This gave an ageing signature consistent with previous analyses. In addition, we performed analyses on tissue-specific subsections of these datasets for brain, heart and muscle revealing some novel tissue specific differences in network connectivity.

## RESULTS

### Most significant ageing gene expression signatures

The global meta-analysis across various tissues in mice, rats and humans identified 449 genes overexpressed with age and 162 underexpressed with age. This is considerably more than the results of de Magalhães, et al. [4], where 56 overexpressed and 17 underexpressed genes were identified. For the tissue-specific analyses, in brain 147 genes were overexpressed and 16 genes were underexpressed, in heart 35 genes were overexpressed and 5 genes were underexpressed, and in muscle 49 genes were overexpressed with 73 genes underexpressed. The top-5 overexpressed genes for each analysis are presented in Table 1 and the top-5 underexpressed genes for each analysis are presented in Table 2.

**Table 1:** Top-5 genes most consistently overexpressed with age across datasets for all tissues and for each tissue studied.

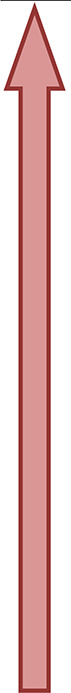	**All Tissues – 449 genes**		
	**Symbol**	**Gene name**	**p-value (1.01e-3)**
	*C1QA*	Complement C1q A chain	3.54e-22
	*GPNMB*	Glycoprotein nmb	1.64e-21
	*B2M*	Beta-2-microglobulin	2.55e-20
	*EFEMP1*	EGF containing fibulin extracellular matrix protein 1	8.06e-20
	*C1QC*	Complement C1q C chain	1.07e-18
	**Brain – 147 genes**		
	**Symbol**	**Gene name**	**p-value (2.95e-4)**
	*C1QA*	Complement C1q A chain	1.21e-15
	*GFAP*	Glial fibrillary acidic protein	1.00e-13
	*C1QB*	Complement C1q B chain	7.06e-13
	*C1QC*	Complement C1q C chain	2.06e-12
	*B2M*	Beta-2-microglobulin	1.19e-11
	**Heart – 35 genes**		
	**Symbol**	**Gene name**	**p-value (6.43e-4)**
	*MGP*	Matrix Gla protein	5.57e-4
	*MAOA*	Monoamine oxidase	5.57e-4
	*CP*	Ceruloplasmin	5.57e-4
	*VAT1*	Vesicle amine transport 1	8.63e-4
	*TMED3*	Transmembrane p24 trafficking protein 3	8.63e-4
	**Muscle – 49 genes**		
	**Symbol**	**Gene name**	**p-value (4.89e-4)**
	*CDKN1A*	Cyclin dependent kinase inhibitor 1A	1.84e-8
	*RNF115*	Ring finger protein 115	7.22e-7
	*EFEMP1*	EGF containing fibulin extracellular matrix protein 1	7.22e-7
	*CHRNA1*	Cholinergic receptor nicotinic alpha 1 subunit	2.57e-6
	*RPS27L*	Ribosomal protein S27 like	4.22e-6

The value given between brackets in the ‘p-value’ column header is the p-value threshold at which FDR <0.05.

**Table 2:** Top-5 genes most consistently underexpressed with age across datasets for all tissues and for each tissue studied.

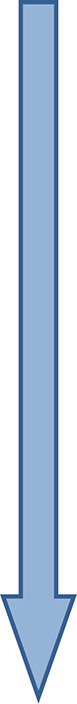	**All Tissues – 162 genes**		
	**Symbol**	**Gene name**	**p-value (7.13e-4)**
	*UQCRFS1*	Ubiquinol-cytochrome c reductase, Rieske iron-sulfur polypeptide 1	1.96e-9
	*SUCLG1*	Succinate-CoA ligase alpha subunit	4.11e-9
	*MLF1*	Myeloid leukemia factor 1	1.37e-8
	*UROS*	Uroporphyrinogen III synthase	4.46e-8
	*FKBP4*	FKBP prolyl isomerase 4	4.58e-8
	**Brain – 16 genes**		
	**Symbol**	**Gene name**	**p-value (4.12e-5)**
	*CX3CL1*	C-X3-C motif chemokine ligand 1	1.23e-8
	*OPCML*	Opiod binding protin.cell adhesion molecule like	2.45e-7
	*SOX11*	SRY-box transcription factor 11	6.97e-7
	*DLG3*	Discs large MAGUK scaffold protein 3	1.13e-6
	*DCLK1*	Doublecortin like kinase 1	3.69e-6
	**Heart – 5 genes**		
	**Symbol**	**Gene name**	**p-value (2.67e-3)**
	*FKBP4*	FKBP prolyl isomerase 4	3.38e-5
	*NDUFS7*	NADH:ubiquinone oxidoreductase core subunit S7	1.79e-3
	*APOOL*	Apolipoprotein O like	2.67e-3
	*OSGEPL1*	O-sialoglycoprotein endopeptidase like 1	2.67e-3
	*KLHL30*	Kelch like family member 30	2.67e-3
	**Muscle – 73 genes**		
	**Symbol**	**Gene name**	**p-value (4.88e-4)**
	*TFRC*	Transferrin receptor	1.78e-9
	*STRADB*	STE20 related adaptor beta	2.88e-8
	*NDUFC1*	NADH:ubiquinone oxidoreductase subunit C1	4.05e-7
	*COL15A1*	Collagen type XV alpha 1 chain	9.30e-7
	*CKMT2*	Creatine kinase, mitochondrial 2	9.30e-7

The value given between brackets in the ‘p-value’ column header is the p-value threshold at which FDR <0.05.

The most significantly overexpressed genes in this meta-analysis were principally involved in immune responses and inflammation, particularly for the global and the brain-specific analyses. Several complement proteins were overexpressed in these analyses, with *C1QA* appearing at the top of both the global and brain-specific analyses, *C1QC* likewise appears in both lists. The top genes in the heart-specific results include the structural protein gene *MGP*, genes involved in amine metabolism and oxidation-reduction processes (*MAOA* and *VAT1*) as well as the iron and copper metabolism gene *CP*. In muscle the top overexpressed gene was *CDKN1A*, a cell cycle regulator. Other interesting genes overexpressed in muscle include *EFEMP1*, a gene involved in eye morphogenesis that has demonstrated involvement in premature-aging like phenotypes in mice, possibly playing a role in fascial structural integrity [6], and that has recently been shown to be overexpressed in aged mouse aorta [7] and *CHRNA1* that codes for a muscle acetylcholine receptor subunit.

A common theme across the top underexpressed genes is mitochondrial metabolism. In the global results, the top underexpressed gene is *UQCRFS1*, a subunit of mitochondrial complex III, while in heart *NDUFS7*, a component of mitochondrial complex I, is the second most significantly underexpressed gene. Another mitochondrial complex I subunit, *NDUFC1* was the third most significantly underexpressed gene in muscle. The brain is the only tissue studied that did not see an underexpression of mitochondrial genes. Indeed, all the top-5 genes underexpressed in the brain signature have clear roles in neuronal signalling and/or development. Complete lists of all significant genes for all the analyses can be found in Supplementary Tables 3–10, while intersections between the results from each analysis can be found in Supplementary Table 27.

Interestingly, several genes with known involvement in ageing-modulating pathways were differentially expressed, for instance *IGF1* was underexpressed, while *IGF2R* and *RICTOR* were overexpressed in the global meta-analysis.

### Comparison with GenAge signature

**Figure 1:**
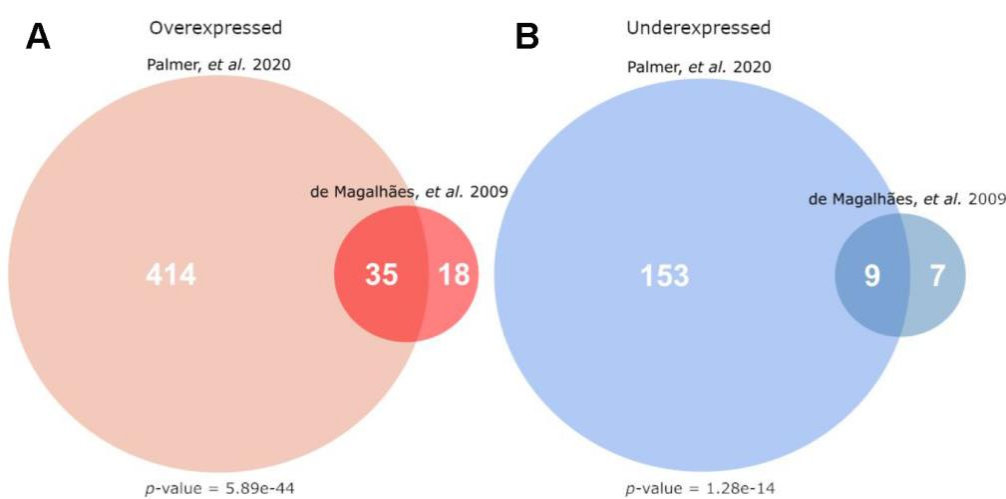
**Overlap of this current work’s meta-analysis (Palmer et al.) with the microarray signature of mammalian ageing currently hosted on GenAge (de Magalhães, et al.)** [4]). (**A**) Gives the overlap for genes overexpressed with age, while (**B**) gives the overlap for genes underexpressed with age. The p-values given are the result of a hypergeometric test, testing the significance of the given overlap using all other protein-coding genes as a background (i.e. all genes not differentially expressed in the direction of the given analysis).

The results from the complete meta-analysis were first compared to the results from the 2009 microarray meta-analysis available on the GenAge database [4]. These two meta-analyses used similar methods, and this new analysis identified 66% and 56.3% of the genes identified previously for over- and underexpressed categories respectively. The overlap for each class of differential expression (over- and underexpressed) between this and the previous meta-analysis are shown in Figure 1.

There was significant overlap between these results and the GenAge signature for both over- (Figure 1A) and underexpressed (Figure 1B) genes (hypergeometric test, *p*<1e-10 for both comparisons), expected given the large overlap of studies included in both analyses.

**Figure 2:**
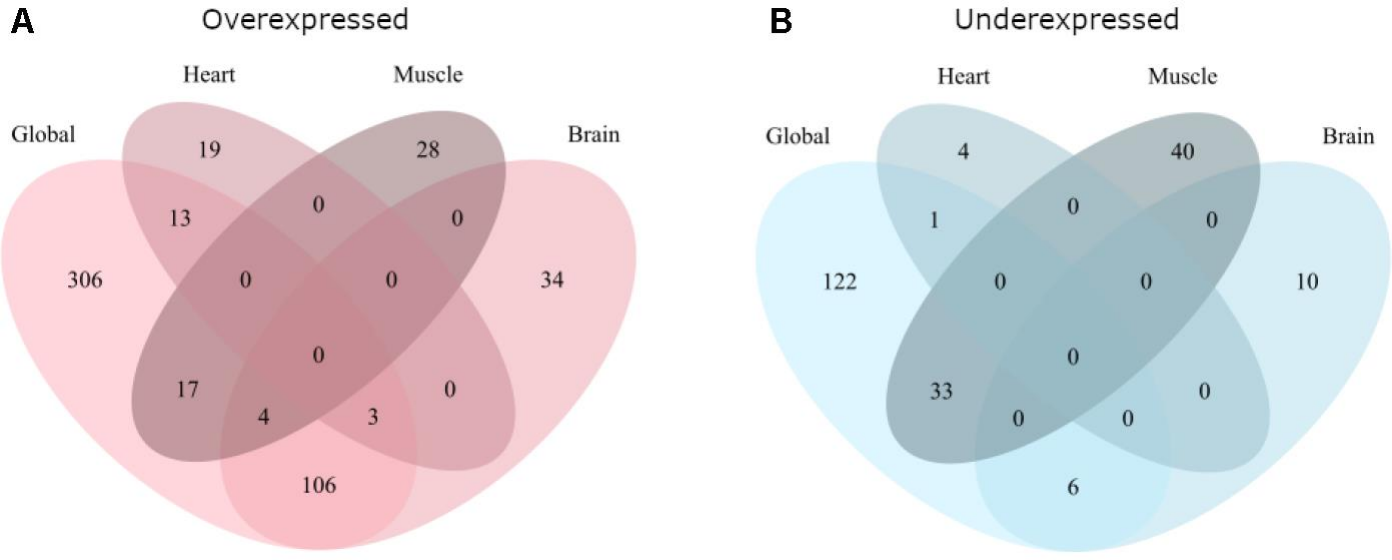
**Overlap of the global and tissue-specific results of this meta-analysis.** (**A**) Gives the overlap for genes overexpressed with age while (**B**) gives the overlap for genes underexpressed with age.

Further, the overlap between the global and tissue-specific analyses was tested for overexpressed and underexpressed genes separately using pairwise hypergeometric tests (Bonferroni corrected). The overlaps between the analyses are shown in Figure 2.

For overexpressed genes (Figure 2A) there was significant overlap between the global analysis and all three tissues (hypergeometric test, *p*<1e-10 for all comparisons). The brain analysis also overlapped significantly with the heart (hypergeometric test, *p*=1.43e-2) and muscle (hypergeometric test, *p*=3.17e-3).

For underexpressed genes (Figure 2B) the global analysis only significantly overlapped with the brain (hypergeometric test, *p*=1.44e-8) and the muscle (hypergeometric test, *p*<1e-10) analyses. No other overlaps were significant.

For both over- and underexpressed genes, there were no genes differentially expressed in all four analyses, nor in both heart and muscle.

### Overlap with other ageing databases

In addition to the GenAge ageing expression signature, this meta-analysis was compared to other gene lists hosted on the Human Ageing Genomic Resources (HAGR). These were the GenAge database of genes suspected to be involved in human ageing [8], the GenDR database of genes differentially expressed with dietary restriction in model organisms [5] and the LongevityMap database of human genes with genetic variants associated with longevity [9].

There was a significant overlap of the genes differentially expressed with age in the complete meta-analysis with both human GenAge genes and the genes with longevity associated variants found in LongevityMap, however there was no overlap with the dietary restriction signature from GenDR, or the human homologues of mouse genes that can modulate longevity in either direction (Table 3).

**Table 3:** Overlap of this current work’s meta-analysis with other relevant gene lists, tested by the hypergeometric test (Bonferroni corrected).

**Database**	**Description**	**Size**	**Overlapping**	***p*-value**
GenDR	Expression signature of dietary restriction in mammals.	158	6	1
GenAge	Curated database of human ageing-related genes.	307	25	1.20e-4
→ Pro-longevity	Human homologues of pro-longevity mouse genes.	80	6	0.290
→ Anti-longevity	Human homologues of anti-longevity mouse genes.	28	3	0.404
LongevityMap	Database of human genetic variants associated with longevity.	358	26	5.74e-4

The overlap shown includes all differentially expressed genes from the expression datasets, regardless of the direction of expression change (611 genes total). Comparisons made are with the GenDR dietary restriction expression signature, the human entries of GenAge which includes genes for which evidence exists of their involvement in ageing, human homologues of genes shown to be pro- or anti-longevity in mice, and genes with longevity associated variants from LongevityMap.

### Functional classification analysis

The detected ageing expression signature was tested for GO enrichment, in addition to the use of data mining methods to identify the most important GO terms that could be used in the assignment of each gene to a differential expression class. The purpose of this dual analysis was to provide functional descriptions from two very different methods, hopefully providing a robust description of functional changes with age.

GO enrichment analysis was performed for each meta-analysis (global, brain, heart, muscle) on the over- and underexpressed expression signatures separately and the significantly enriched GO terms were ranked by *p*-value (Supplementary Tables 11–18).

The machine learning analysis was likewise conducted on each tissue, and the GO terms determined to be predictive of each expression class (overexpressed, underexpressed or unchanged) were ranked in terms of descending average probability (Supplementary Tables 19–26).

To provide a comprehensive picture of the functional changes associated with the ageing expression signature, top-ranked terms that overlap between these two analyses are presented below for GO terms associated with overexpressed (Table 4) and underexpressed genes (Table 5), for each tissue. The criteria for inclusion in these tables is that the term was significantly differentially expressed (*p*<0.05) and present in the top-20 terms for the prediction of the given expression class. The data mining precision was prioritised over enrichment significance, and so they have been ranked in the following tables according to their precision value. Note that although many of the precision values for the top-ranked terms are relatively low, they are much higher than the class label’s relative frequency (given in the column header), which is the precision that a classifier would get by randomly classifying the genes.

**Table 4:** Summary of results from the GO enrichment and feature importance analyses on the genes overexpressed with age.

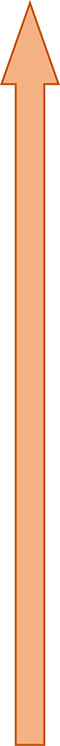	**All tissues (Overexpressed)**			
	**GO.ID**	**Term**	***p*-value**	**Precision (0.0251)**
	GO:2001198	Regulation of dendritic cell differentiation	2.00e-4	0.613
	GO:0071276	Cellular response to cadmium ion	2.90e-5	0.571
	GO:0071294	Cellular response to zinc ion	3.00e-6	0.452
	GO:0006958	Complement activation, classical pathway	1.30e-6	0.276
	O:0051043	Regulation of membrane protein ectodomain proteolysis	3.30e-4	0.267
	**Brain (Overexpressed)**			
	**GO.ID**	**Term**	***p*-value**	**Precision (0.0083)**
	GO:2001198	Regulation of dendritic cell differentiation	1.40e-4	0.298
	GO:0006958	Complement activation, classical pathway	3.30e-7	0.135
	GO:0071803	Positive regulation of podosome assembly	7.90e-5	0.128
	GO:1902106	Negative regulation of leukocyte differentiation	2.20e-4	0.118
	GO:0032570	Response to progesterone	9.30e-4	0.109
	**Heart (Overexpressed)**			
	**GO.ID**	**Term**	***p*-value**	**Precision (0.0025)**
	GO:0071295	Cellular response to vitamin	2.56e-2	0.0761
	GO:0042246	Tissue regeneration	5.90e-3	0.0462
	GO:0055072	Iron ion homeostasis	4.49e-2	0.0323
	GO:0007205	Protein kinase C-activating G protein-coupled receptor signalling pathway	1.90e-3	0.0289
	GO:0018149	Peptide cross-linking	5.70e-3	0.0285
	**Muscle (Overexpressed)**			
	**GO.ID**	**Term**	***p*-value**	**Precision (0.0028)**
	GO:0031571	Mitotic G1 DNA damage checkpoint	2.70e-4	0.08
	GO:0032925	Regulation of activin receptor signalling pathway	4.21e-2	0.0566
	GO:0006195	Purine nucleotide catabolic process	6.99e-3	0.0279
	GO:0042771	Intrinsic apoptotic signaling pathway in response to DNA damage by p53 class mediator	2.70e-4	0.0251
	GO:2000379	Positive regulation of reactive oxygen species metabolic process	5.03e-3	0.025

Presented here are a selection of terms for each tissue which were both significantly enriched in the given gene list and present in the top-20 terms, ranked by precision, for the prediction of a gene as being overexpressed by the Random Forest model. The value given between brackets in the Precision column header is the class label’s relative class frequency, i.e. the precision that a classifier would get by randomly classifying the genes.

**Table 5:** Summary of results from the GO enrichment and feature importance analyses on the genes underexpressed with age.

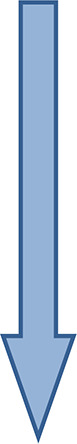	**All tissues (Underexpressed)**			
	**GO.ID**	**Term**	***p*-value**	**Precision (0.0090)**
	GO:0010510	Regulation of acetyl-CoA biosynthetic process from pyruvate	3.20e-6	0.374
	GO:0006122	Mitochondrial electron transport, ubiquinol to cytochrome c	1.60e-9	0.356
	GO:0006099	Tricarboxylic acid cycle	1.50e-10	0.281
	GO:0006107	Oxaloacetate metabolic process	1.20e-4	0.217
	GO:0007528	Neuromuscular junction development	3.06e-2	0.204
	**Brain (Underexpressed)**			
	**GO.ID**	**Term**	***p*-value**	**Precision (0.0008)**
	GO:0021782	Glial cell development	3.69e-2	0.0489
	GO:0021510	Spinal cord development	4.20e-2	0.0425
	**Muscle (Underexpressed)**			
	**GO.ID**	**Term**	***p*-value**	**Precision (0.0041)**
	GO:0043455	Regulation of secondary metabolic process	1.16e-3	0.203
	GO:0006094	Gluconeogenesis	1.50e-9	0.170
	GO:0061621	Canonical glycolysis	4.30e-6	0.146
	GO:0042776	Mitochondrial ATP synthesis coupled proton transport	4.90e-5	0.119
	GO:0006107	Oxaloacetate metabolic process	9.70e-4	0.103

Presented here are a selection of terms for each tissue which were both significantly enriched in the given gene list and present in the top-20 terms, ranked by precision, for the prediction of a gene as being underexpressed by the Random Forest model. The value given between brackets in the Precision column header is the class label’s relative class frequency, i.e. the precision that a classifier would get by randomly classifying the genes. It should be noted that the list of genes underexpressed in the heart was too small for a meaningful analysis and so has been left out.

Terms describing the overexpressed genes were predominantly related to immune responses; for instance, “Regulation of dendritic cell differentiation” was the best predictor of overexpression in both the global and brain analyses, with an average precision of 0.613 and 0.298 respectively, while also being significantly enriched in both cases. Likewise, “Complement activation, classical pathway” another immune term was highlighted in both these analyses, while in brain “Positive regulation of podosome assembly” and “Negative regulation of leukocyte differentiation” were both identified strongly by both analysis methods.

Another theme amongst the overexpressed genes that crosses tissues is cellular response functions, particularly in relation to stress, for instance terms raised by the global analysis include “Cellular response to cadmium ion” and “Cellular response to zinc ion”, while in heart “Cellular response to vitamin” and “Iron ion homeostasis” were identified, and finally in muscle “Positive regulation of reactive oxygen species metabolic process” was determined to be of interest.

Terms describing the underexpressed genes were less precise and in lower number than those describing overexpressed genes due to the lower numbers of underexpressed genes overall (excepting muscle). The global meta-analysis is dominated by metabolic and developmental terms, with the metabolic theme being shared with muscle (e.g. “Oxaloacetate metabolic process” was considered important in both) while the developmental theme was shared with the brain. Interestingly, the machine learning and enrichment analyses shared little specific agreement regarding genes underexpressed in the brain, with only two terms being agreed on as interesting by both methods, this is likely due to the low number of genes underexpressed in the brain (16).

### Tissue specificity of the ageing transcriptome

To determine if there was an association between tissue specificity and the ageing expression signature, the τ index of tissue specificity was calculated for all genes studied in the meta-analysis, using the expression data from the GTEx project. This yielded a bimodal distribution of gene specificity, typical of this measure (Supplementary Figure 2).

There was a weak negative association detected between differential expression with age and high (τ>0.8) tissue specificity for overexpressed genes in the global (*p*<1e-10, chi-squared; phi=-0.87) and brain (*p*=2.59e-7; phi=-0.042) analyses, and for underexpressed genes in the global (*p*=6.43e-7, phi=-0.041) and muscle (*p*=1.84e-4, phi=-0.033) analyses. Complete analysis and median tau values are presented in Supplementary Table 2.

### Network analysis of ageing signatures

For the protein-protein interactions (PPI) network (Supplementary Figure 3A), degree centrality (Supplementary Figures 4A–7A, 8) was higher for genes over- (median=1.06e-3) and underexpressed (median=1.43e-3) in the global analysis when compared to unchanged (median=6.23e-4) genes (*p*<1e-10 and *p*=4.3e-10, respectively). The muscle signature showed the same result for overexpressed genes (median=1.37e-3) although degree was lower (median=9.97e-4) in genes underexpressed with age in the muscle compared to unchanged (median=6.23e-4) genes (*p*=0.026 and *p*=0.013, respectively). Interestingly, degree centrality was borderline significantly higher in overexpressed genes (median=9.35e-4) compared to unchanged genes (median=6.23e-4) in the brain (*p*=0.048), but there was no such difference for genes underexpressed in the brain. The heart signature showed no difference in degree centrality, or indeed any other centrality measure.

Betweenness centrality in the PPI network (Supplementary Figures 4B–7B, 9) saw a very similar pattern. As with degree, betweenness was higher in genes both over- (median=2.8e-5) and underexpressed (median=4.57e-5) in the global analysis when compared to unchanged (median=8.59e-6) genes (*p*<1e-10 and *p*=1.9e-10, respectively), as well as being higher in both over- (median=3.5e-5) and underexpressed (median=2.29e-5) genes, compared to unchanged (median=9e-6) in the muscle (*p*=0.0138 and *p*=5.7e-3, respectively). Again, betweenness was also higher in genes overexpressed (median=2.5e-5) compared to unchanged (median=9.02e-6) in the brain (*p*=3.3e-4), but there was no change in genes underexpressed in the brain.

Closeness centrality in the PPI network (Supplementary Figures 4C–7C, 10) was higher in both over- (median=0.331) and underexpressed (median=0.334) genes in the global analysis compared to unchanged (median=0.321) genes (*p*=2.7e-10 and *p*=4.3e-6, respectively), however this was not observed in any other signature, and the increase in the global signature was very small.

For the co-expression network (Supplementary Figure 3B), degree centrality (Supplementary Figures 11A–14A, 15) was lower in genes underexpressed in the global (*p*=1.7e-3, median=9.47e-4) and muscle (*p*=0.025, median=9.92e-4) analyses compared to unchanged genes (median=2.08e-3), yet in the brain analysis degree was higher in the underexpressed genes (median=1.59e-2) compared to either overexpressed (*p*=8.8e-4, median=2.08e-3) or unchanged genes (*p*=5.81e-3, median=2.08e-3).

Betweenness centrality in the co-expression network (Supplementary Figures 11B–14B, 16) was only changed in the brain signature, where, as with degree, the underexpressed genes (median=3.98e-4) had a higher betweenness than unchanged genes (*p*=0.034, median=7.88e-4), although in this case there was no significant difference between over- and underexpressed genes.

Finally, closeness centrality in the co-expression network (Supplementary Figures 11C–14C, 17) was lower in both over- (p<1e-10, median=0.154) and underexpressed (*p*=2.1e-4, median=0.152) genes relative to unchanged genes (median=0.163) in the global analysis as well as in overexpressed genes in the heart analysis (median=0.145) when compared to unchanged genes (*p*=4.2e-4, median=0.163) and underexpressed genes in the muscle analysis (median=0.151) when compared to unchanged genes (*p*=1.3e-3, median=0.163). In the brain analysis, closeness was lower in the overexpressed genes (median=0.154) compared to both unchanged (*p*=1.1e-5, median=0.163) and underexpressed (*p*=2.8e-5, median=0.187) genes, while the underexpressed genes also had higher closeness compared to the unchanged genes (*p*=0.021).

### Evolutionary conservation of ageing signature genes

There were no significant differences between dN/dS ratios (the ratio of nonsynonymous to synonymous substitutions between the species) of genes over- or underexpressed with age when compared to either unchanged genes or to the opposite expression category, for either human-mouse or human-rat ratios (Supplementary Figures 18, 19). The median values tended towards a lower dN/dS in those genes underexpressed with age relative to those overexpressed with age, with the median dN/dS being 0.096 and 0.093 in underexpressed genes and 0.12 and 0.11 in overexpressed genes for human-mouse and human-rat comparisons, respectively.

## DISCUSSION

There was a significant overlap between this meta-analysis and the results of de Magalhães, et al. [4] (Figure 1) for both over- and underexpressed genes. This overlap, although significant, is not as extensive as might have been expected, potentially due to the differing biases in microarray and RNA-Seq results [10], or the heterogeneity demonstrated in expression patterns of the mammalian immune response [11]. Despite this, the functional themes of the detected genes were much the same with overexpressed genes being broadly immune and underexpressed genes being broadly metabolic.

Enrichment analysis was coupled with data mining to identify GO terms that robustly describe the processes associated with the altered genes. Examining the top-ranked GO terms that these methods agreed on (Tables 4 and 5) reveals some interesting differences and similarities between the studied tissues. The global analysis of 127 datasets is typical of previous large-scale expression studies and meta-analyses [4, 12, 13], showing overexpression of immune genes, stress responses and proteolysis (Table 4A), as well as underexpression of metabolic and energy metabolism. The preponderance of inflammatory and stress response genes in particular is reminiscent of the inflammageing hypothesis [14], which argues that ageing is caused by steadily failing responses to stress, in particular responses to the increased antigenic load that comes with age. Coupled with the overexpression of immune and inflammatory genes, the underexpression of metabolic genes is implicated not just in ageing, but in several ageing-related diseases for instance Alzheimer’s [15] and Duchenne muscular dystrophy [16].

A similar profile was seen in the brain with immune categories dominating the top-ranked terms, including “Regulation of dendritic cell differentiation”, which was also the most predictive GO term of overexpression with age in the global analysis. There is some evidence suggesting a causative role of immune processes in brain ageing, for example astrocytosis, abnormal proliferation of the cells responsible for (among many other functions) regulation of inflammation in the central nervous system [17] is associated with loss of myelin in Alzheimer’s disease, Parkinson disease and ageing [18]. It is possible that changes between different brain regions exist that could not be detected due to the study design. Indeed different regions of the brain do appear to suffer age-related decline at different rates [19].

Differential ageing between tissues was seen in the other analyses as well, and it is unclear to what extent tissues age at the same rate. Epigenetic measures have shown some minor differences in the rate of ageing between breast and other tissues [20], and environmental effects accelerate age-related changes in exposed tissues, for instance skin ageing is influenced by smoking [21] and air pollution [22]. The extent to which such changes can be considered increases in the rate of ageing are suspect however [23], it could simply be that extrinsic stressors cause damage similar to that of ageing. The data presented here suggest some differences in transcriptomic ageing between tissues, particularly between the overexpressed signatures of the brain and the heart/muscle, with the brain showing changes in immune categories while the heart and muscle show changes in local homeostasis and protein catabolism (Table 4).

These categories are consistent with previous analyses of ageing transcription signatures. de Magalhães, et al. [4] likewise identified several overexpressed immune and xenobiotic terms, with metabolic terms being enriched in the underexpressed genes; while the more recent GTEx consortium analysis of human ageing has also reported that genes underexpressed with age in multiple tissues are consistently enriched for metabolic, in particular mitochondrial, GO terms [12].

An interesting result was the significant underexpression of some immune genes (*MLF1*, *FKBP4*) in the meta-analysis (Table 2A). Dysregulation of the immune system may in part explain why the immune response becomes less effective with age, indeed old mice have been shown to have increased heterogeneity of transcriptional response to immune stimulus in their CD4^+^ T cells, with results suggesting that they are less able to upregulate adaptive response programs when necessary [24].

Of the other HAGR databases tested, GenDR and the longevity modulating mouse genes from GenAge did not show a significant overlap (Table 3). This is possibly due to the inclusion of human data in this meta-analysis, whereas the dietary restriction signature hosted on GenDR is based on mouse, rat and pig [5], and the longevity modulating mouse genes may not always be transferable to other species, or necessarily be differentially expressed. Alternatively, although dietary restriction slows ageing, it may do so by affecting pathways that are not commonly altered with age and that perhaps modulate ageing at a deeper level. While there is evidence that dietary restriction is able to reverse many ageing transcriptional changes [25, 26], it appears that the lifespan extension may be caused by an upregulation of stress responses and repair mechanisms [27] and thus dietary restriction may combat ageing by improving defenses to ageing-related damage, rather than altering the ageing processes themselves. Additionally, dietary restriction may weaken the adaptive immune system in aged organisms [28], whereas the opposite might be expected if it were simply reversing or slowing ageing processes.

The significant overlap between the ageing expression signature and both GenAge and Longevity Map is interesting because the genes recorded in those databases are genes with either evidence of involvement in ageing or genes with genetic association to longevity, neither of which would necessarily be expected to be altered with age. One caveat is that a large number of immune genes were identified in these expression signatures, and several of the largest contributing studies in LongevityMap were explicitly studying variation in immune genes and how it affects ageing, as such LongevityMap would be expected to skew towards immune and inflammation genes.

These data suggest the most detectable ageing expression changes are those that occur in genes expressed across tissues, with a weak negative association observed between genes being tissue specific (τ>0.8) and being differentially expressed with age for overexpressed genes in the global and brain analyses, and underexpressed genes in the global and muscle analyses (Supplementary Table 2). This result is corroborated by other studies, for instance in mice genes differentially expressed with age tend to be differentially expressed across multiple tissues, although gene expression changes in some tissues, for example the liver, do tend to be more tissue-specific [29]. Further, the AGEMAP project was able to cluster tissues into three modes of ageing: neural, vascular and steroid responsiveness [30]. This suggests that while there may be distinct ageing transcriptional profiles between tissues, there are sets of tissues which age by similar mechanisms, with similar expression changes. It should be noted that the nature of this meta-analysis means that only the most consistently differentially expressed genes were detected. As such there is potentially a bias towards genes that are both highly expressed and expressed across tissues, since these will have been detected in more studies.

Interestingly, while the underexpressed signatures focused on metabolic and developmental genes, both heart and muscle showed distinct overexpressed signatures relative to the similar profiles observed in the global and brain analyses. The heart, for instance, shows a focus on cellular responses including to vitamin and iron homeostasis (Table 4C). Iron homeostasis deregulation with age has been shown to occur in several tissues and is a possible driver of oxidative stress in aged tissues, with the activation of iron detoxification proteins being a possible adaptive measure to such changes [31]. The muscle shows overexpression of cell-cycle mediators (Table 4D), which while typically associated with cellular senescence and the prevention of cancer, are also involved in the repair of DNA damage, apoptosis, autophagy, immune responses and metabolism [32]. Indeed, apoptosis in skeletal muscle may be one of the causes of fiber loss that results in sarcopenia [33].

Considering the PPI network, the higher degree centrality of genes differentially expressed with age in most tissues is not especially surprising. Several of the identified genes are well studied and PPI data favours proteins of high abundance [34] and with high publication coverage [35]. Despite this, coupling the higher degree centrality with the higher betweenness centrality seen in the same tissues (Supplementary Figures 8, 9), and the higher closeness centrality seen in differentially expressed genes from the global analysis (Supplementary Figure 10) there is evidence that genes differentially expressed with age tend to be highly connected within PPI networks, suggesting possible regulatory roles and are thus potential bottlenecks to the flow of information through the network, making them interesting targets for intervention to study the regulation of these networks.

In the co-expression network, degree centrality (Supplementary Figure 15) was lower in underexpressed genes in the global and muscle analyses, yet it was higher in underexpressed genes in the brain analysis. This trend was mirrored by betweenness centrality (Supplementary Figure 16), which was higher in genes underexpressed in the brain despite not being changed in any other signature. Likewise, while closeness centrality tended to be lower in both over- and underexpressed genes across the analyses (Supplementary Figure 17) it was higher in genes underexpressed in the brain. The high centrality of both over- and underexpressed genes in the PPI network, but particularly the high centrality of the underexpressed brain genes in the co-expression network, is interesting since high centrality in biological networks can indicate importance in disease with highly central genes potentially having dramatic or even lethal effects when targeted [36]. Further, co-expression in the brain is disrupted by diseases such as Alzheimer’s disease [37], making these genes potentially important in the pathogenesis of aging brain disease.

To summarise: 1) the ageing expression signature in humans, mice and rats can be predominantly described as an overexpression of genes associated with immune, stress and proteolytic processes coupled with an underexpression of genes associated with metabolic, particularly mitochondrial, and development processes; 2) genes differentially expressed with age tend to be more highly connected in the protein-protein network, particularly in the global and brain signatures; 3) genes underexpressed with age in the brain are highly central in the co-expression network, suggesting these underexpressed genes may have significant effects and, we hypothesize, play a role in cognitive ageing and; 4) the most detectable genes differentially expressed with age tend to be expressed across a broad range of tissues.

We provide the differential expression results used in the meta-analysis (Supplementary Datasets), along with the tau scores (Supplementary Table 2) of tissue specificity calculated from the GTEx database as a resource for the community. These data will be most useful as a validation dataset, reflecting as they do the most commonly observed genes differentially expressed with age, however they may also prove useful for further discovery, for instance as features for further data mining studies, combining these annotations with other databases or fresh experimental data.

## MATERIALS AND METHODS

### Preparation of the dataset

In total, 127 datasets were downloaded from AGEMAP [30] and the Gene Expression Omnibus (GEO) [38] (Supplementary Table 1), covering a total of 37 tissues and cell types. AGEMAP contains the results of microarray experiments on mice at various ages, while the GEO datasets downloaded were identified using the search string:

“((“age”[Subset Variable Type]) or “development stage”[Subset Variable Type]) and “mammals” [organism]”,

returning 335 microarray and RNA-Seq datasets. These were manually filtered to remove non-single channel arrays, single-pathway arrays as well as species that were not of interest. Mutant or diseased samples were likewise removed. Next, RNA-Seq datasets containing raw reads were normalised as reads per kilobase million (RPKM), and all datasets were log2 transformed, if they were not supplied so already.

Linear regression was carried out on each dataset to determine differential expression with age (Equation 1) where Y_ij_ is the expression level of gene j in sample i, Age_i_ is the age at which sample i was taken and ϵ_ij_ is the error term. Coefficients β_0_ and β_1_ were estimated by least squares, and significance was calculated using an F-test.

$$Y_{ij}=\beta_{0j}+\beta_{1j}Age_{i}+\epsilon_{ij}$$ (1)

A cumulative binomial test was then used to identify genes that were significantly differentially expressed across the datasets, taking the probability of success as the probability that any gene was not over-/underexpressed in any dataset, the number of trials as the number of datasets in which the given gene was detected, and the number of successes as the number of datasets in which the given gene was not detected as significant. Thus the test asks, “for a gene; given the number of times a gene was tested across all the data sets, the number of times a gene was significantly differentially expressed across all the data sets, and the probability of seeing any gene differentially expressed, what is the probability that this gene is differentially expressed more than we expect to see by chance?”. False discovery rate (Q) was controlled by randomising the datasets 10,000 times, repeating the analyses with these randomised data, and then carrying out a linear regression on the simulated results to estimate the *p*-value cut-off at which Q<0.05.

The meta-analysis was repeated three times, using only the datasets from specific tissues. Thus, four analyses were carried out, a global analysis of all tissues (127 datasets) and tissue-specific analyses of brain (29 datasets), heart (9 datasets) and muscle (26 datasets).

A summary of the method is given in Supplementary Figure 1.

### Determination of tissue specificity

The expression data from version 7 of the GTEx project [39] was downloaded and used to calculate a τ index for each gene. The τ index is an indicator of how specifically or broadly expressed a gene is, with a τ of 1 indicating expression specific to only one tissue, and a τ of 0 indicating equal expression across all tissues [40]. The τ index for a given gene can be calculated as shown in Equation 2, where N is the number of tissues being studied and x_i_ is the expression profile component for a given tissue, normalised by the maximal component value for that gene (i.e. the expression of that gene in the tissue it is most highly expressed in).

$$\tau =\frac{\sum_{i=1}^{N}\left(1-x_{i}\right)}{N-1}$$ (2)

### Analysis of differentially expressed genes

### Comparison with relevant ageing gene lists

The overlap between the global signature and relevant ageing gene lists was tested using the hypergeometric test [41] with all the genes included in the meta-analysis as the background set. When comparing to the GenAge expression signature, over- and under-expressed genes were considered separately. Comparison to the other Human Ageing Genomic Resource (HAGR) databases (human genes from GenAge [8], GenDR [5] and LongevityMap [9]) was performed ignoring the direction of expression change (Bonferroni corrected).

### Tissue specificity of ageing genes

The association between differential expression with ageing according to the meta-analysis and tissue specificity (defined as a having τ index of >0.8 based on the GTEx data) was tested using a chi-squared test and the phi coefficient was calculated to indicate the strength of the correlation. Association was tested for both over- and underexpressed genes, for all four meta-analyses (Bonferroni corrected).

### Enrichment analysis

The topGO package (v2.28.0) [42] was used in the R programming environment using the weight01 algorithm [43] and Fisher’s exact test to calculate enrichment of GO terms. Genes were mapped to the GO-2017-03-29 release since this is the release utilised by the GO.db package version in Bioconductor 3.5 [44].

### Rule-based precision analysis

To complement the enrichment analysis, Random Forest (RF) machine learning models were used to identify the most important GO terms for the classification of genes as over- or underexpressed with age. The RF algorithm builds many Random Trees (RT) during its training (model construction) phase. Each node in a RT contains a condition that splits the instances (the genes) into two subsets according to the values of the selected feature (in our case, the presence or absence of a GO term in a gene), creating two child nodes. The RF algorithm aims to select features that best split genes (based on their change in expression label) into the two groups, so that genes of different class labels (over vs. under-expressed) are assigned as much as possible to different groups. Next, the algorithm re-runs the previously described split procedure in the two newly generated groups until some user-defined condition is met.

To predict the class label of an unseen gene, for every RT, the conditions in the tree (starting in the root node) are matched against the gene’s features (GO terms from GO-2017-03-14) until a leaf node is reached. When the instance (gene) reaches a leaf node, the most frequent class in the node is selected to be the prediction of the tree. The final prediction of the whole RF model is defined by the simple voting of all RTs.

We used Rule-Based Precision (RBP) [45] to measure the importance of features used by the model. Briefly speaking, to measure the RBP we build several RFs, where each of them in turn comprises many RTs. For each tree and feature (a GO term), we identify all paths in the decision trees from root to leaf that use the positive value of the GO term feature, that is, paths in the tree that “capture” a gene only if the GO term annotates that gene. Then, the method calculates the overall precision of these paths, and uses this precision to rank the GO terms regarding predictive power. The main motivation for using the RBP measure is that it was designed specifically to reward “positive” feature values (GO term annotations), rather than “negative” feature values (lack of GO term annotations), since the former are more reliable. Actually, a negative feature value denotes lack of evidence, rather than evidence for the absence of a given gene function.

### Network analysis

The human PPI network was downloaded from BioGRID version 3.3.123 [46] and non-physical interactions were removed, leaving 219,240 interactions. Additionally, an unweighted co-expression network of highly correlated genes from the GeneFriends RNA-seq co-expression map (V3.1) was also used [47]. The betweenness, closeness and degree (normalised by dividing by the maximum degree of a graph n-1, where n is the number of nodes in graph G) of each gene in these networks were calculated using the ‘networkx’ Python library [48], and the average betweenness, closeness and degree of the genes in each expression signature was determined. The centrality measures of over- and underexpressed genes were then compared to their opposite category, as well as the non-differentially expressed genes by pairwise Mann-Whitney U tests (Bonferroni corrected).

### dN/dS analysis

To identify any differences in the evolutionary conservation of genes differentially expressed with age, the dN/dS ratios for comparison between humans and mice, and humans and rats were obtained from Ensembl Biomart release 96, keeping only those genes with 1 to 1 ortholog homology type between the relevant species and high orthology confidence. These dN/dS ratios compare the rates of synonymous and nonsynonymous substitutions between species for a given gene, giving an idea of the type of selection that gene may be under, if any [49]. The distribution of dN/dS scores was compared by pairwise Mann-Whitney U tests (Bonferroni corrected) across all comparisons between genes overexpressed with age, underexpressed with age and unchanged with age.

### Data availability

The data that supports the findings of this study are available in the supplementary material of this article, which are available on the Integrative Genomics of Ageing Group AgeingSignatures2020_supplementary GitHub repository (https://github.com/maglab/AgeingSignatures2020_supplementary). These data were derived from the resources listed in Supplementary Table 1.

## Supplementary Material

Supplementary Figures

Supplementary Table 1

Supplementary Table 2

Supplementary Tables 3 to 6

Supplementary Tables 7 to 10

Supplementary Tables 11 to 18

Supplementary Tables 19 to 26

Supplementary Table 27
