# Supplementary material for: Ageing transcriptome meta-analysis reveals similarities and differences between key mammalian tissues
Source: Aging (Albany NY). 2021 Feb 11;13(3):3313–41. doi: 10.18632/aging.202648 (PMC7906136; doi:10.18632/aging.202648)
Supplement: Supplementary Figures [file aging-13-202648-s001.pdf]

SUPPLEMENTARY FIGURES

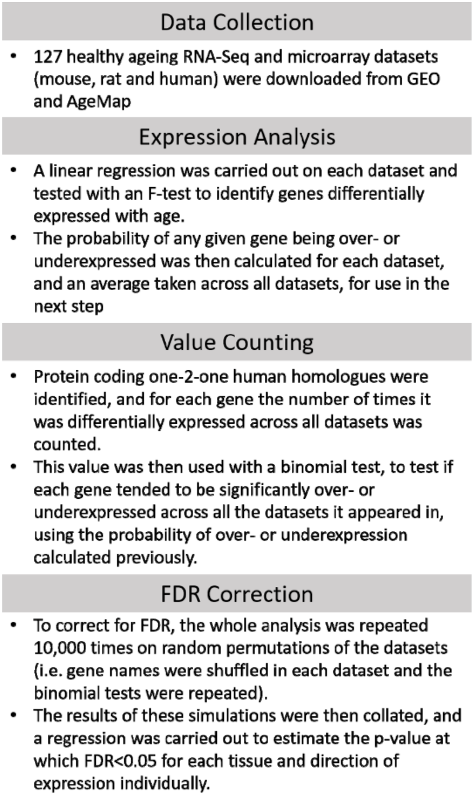

Supplementary Figure 1. Summary of the meta-analysis method.

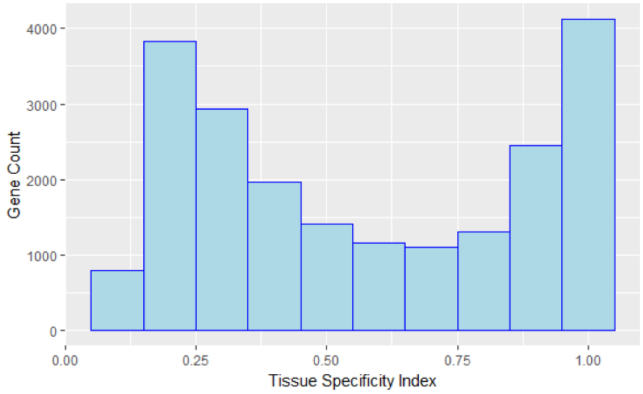

Supplementary Figure 2. Distribution of τ tissue specificity scores in the whole GTEx expression dataset. A τ specificity index of 0 indicates complete nonspecific expression while an index of 1 indicates completely specific expression.

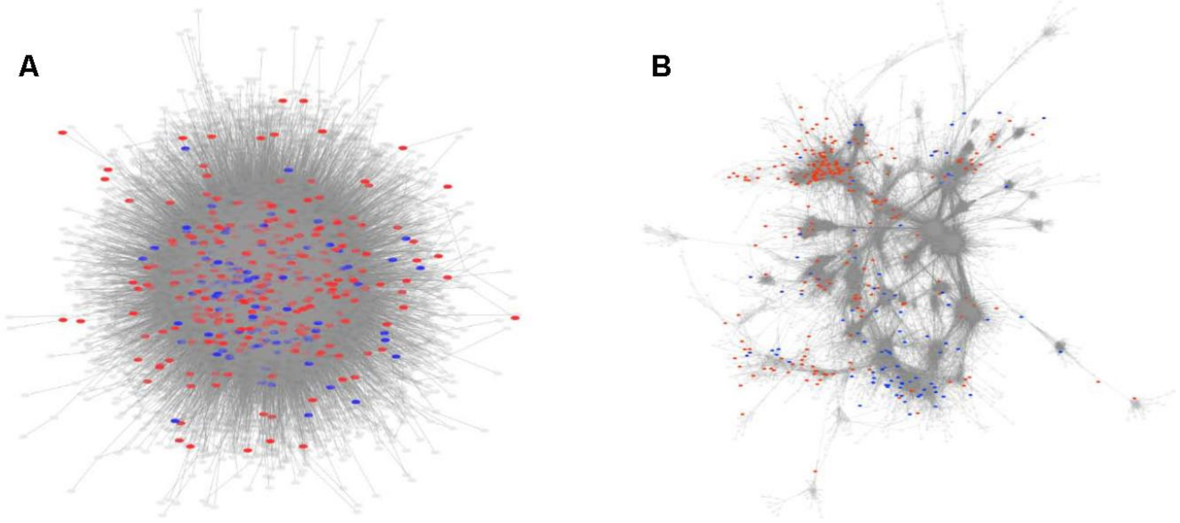

Supplementary Figure 3. Interaction networks showing the locations of the overexpressed (red) and underexpressed (blue) genes in the (**A**) BioGRID PPI network and the (**B**) GeneFriends coexpression network.

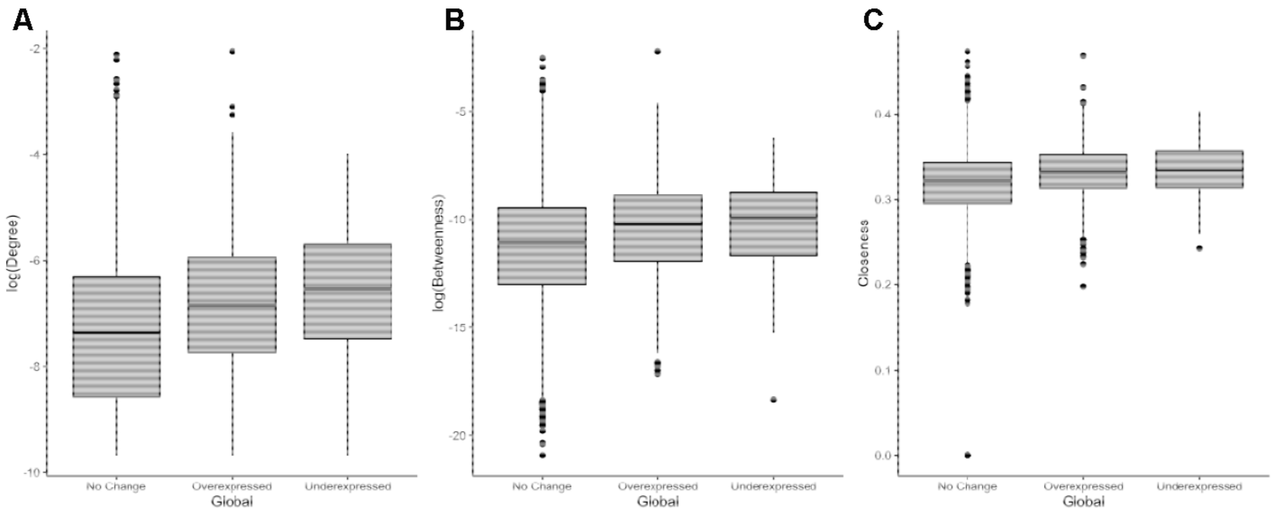

Supplementary Figure 4. Distributions of degree (normalised by dividing by the maximum degree of a graph n-1, where n is the number of nodes in graph G) (**A**), betweenness (**B**) and closeness (**C**) centrality measures in a genome-wide PPI network for overexpressed, underexpressed and unchanged genes from the global analysis

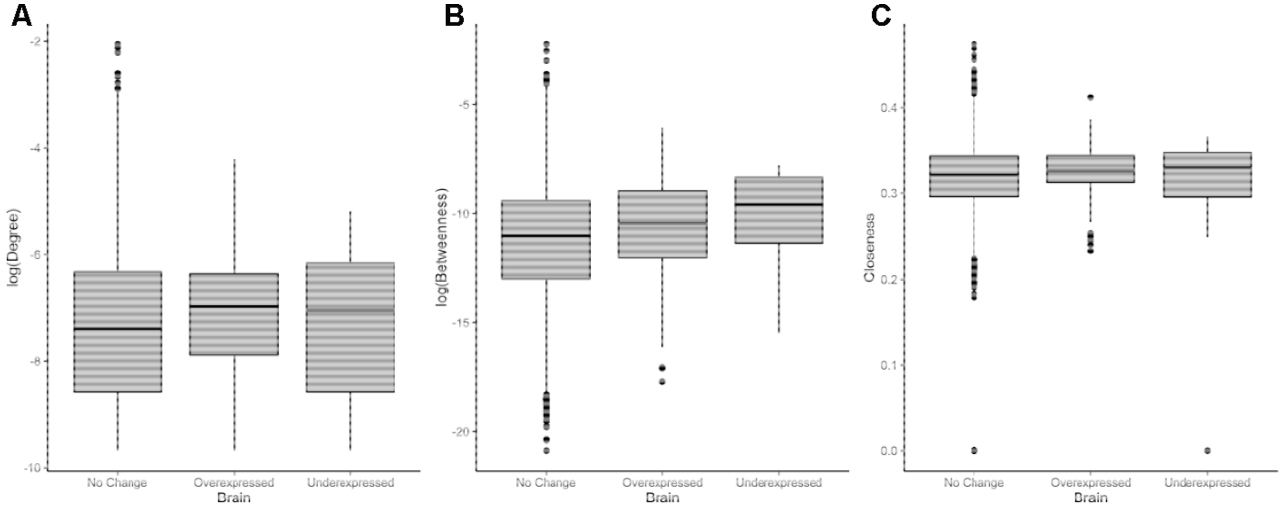

Supplementary Figure 5. Distributions of degree (normalised by dividing by the maximum degree of a graph n-1, where n is the number of nodes in graph G) (**A**), betweenness (**B**) and closeness (**C**) centrality measures in a genome-wide PPI network for overexpressed, underexpressed and unchanged genes from the brain analysis.

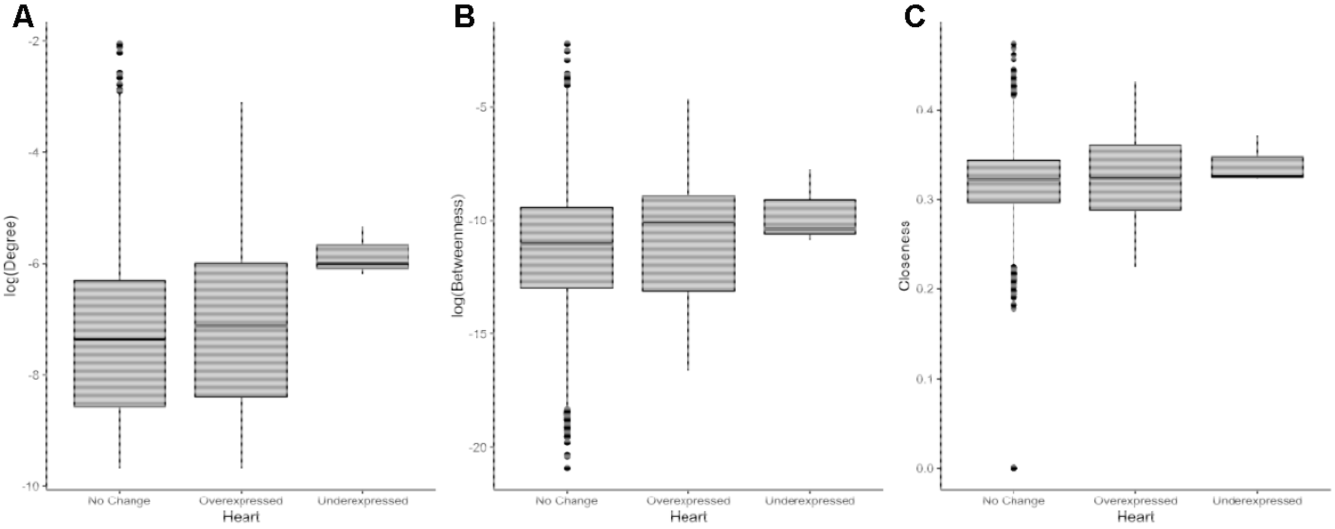

Supplementary Figure 6. Distributions of degree (normalised by dividing by the maximum degree of a graph n-1, where n is the number of nodes in graph G) (**A**), betweenness (**B**) and closeness (**C**) centrality measures in a genome-wide PPI network for overexpressed, underexpressed and unchanged genes from the heart analysis.

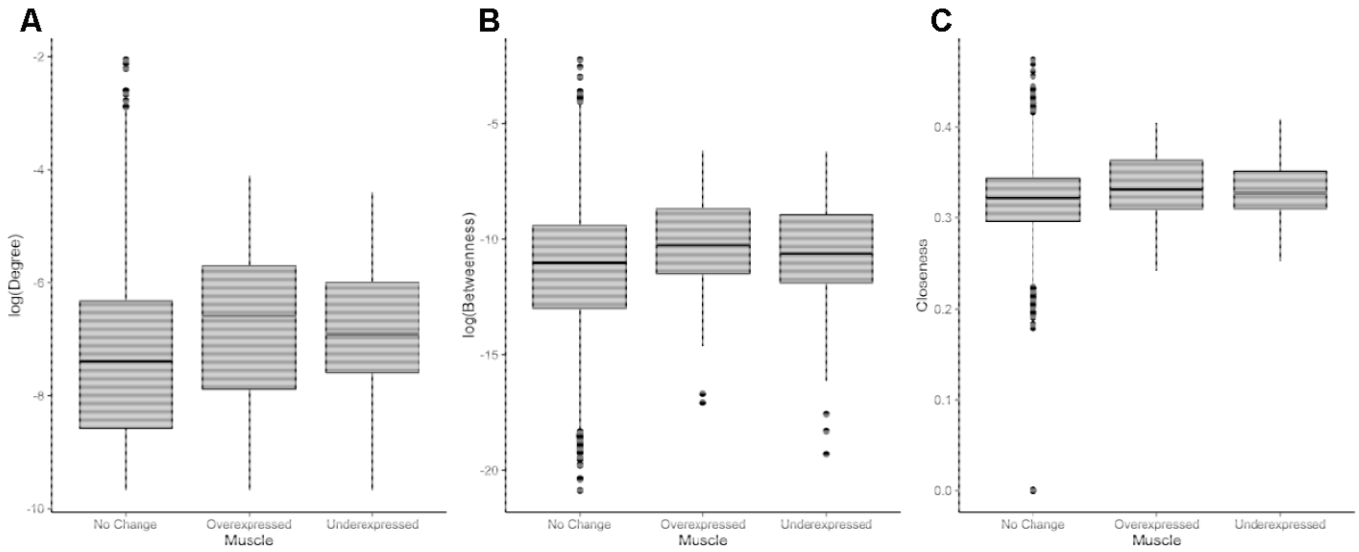

Supplementary Figure 7. Distributions of degree (normalised by dividing by the maximum degree of a graph n-1, where n is the number of nodes in graph G) (**A**), betweenness (**B**) and closeness (**C**) centrality measures in a genome-wide PPI network for overexpressed, underexpressed and unchanged genes from the muscle analysis.

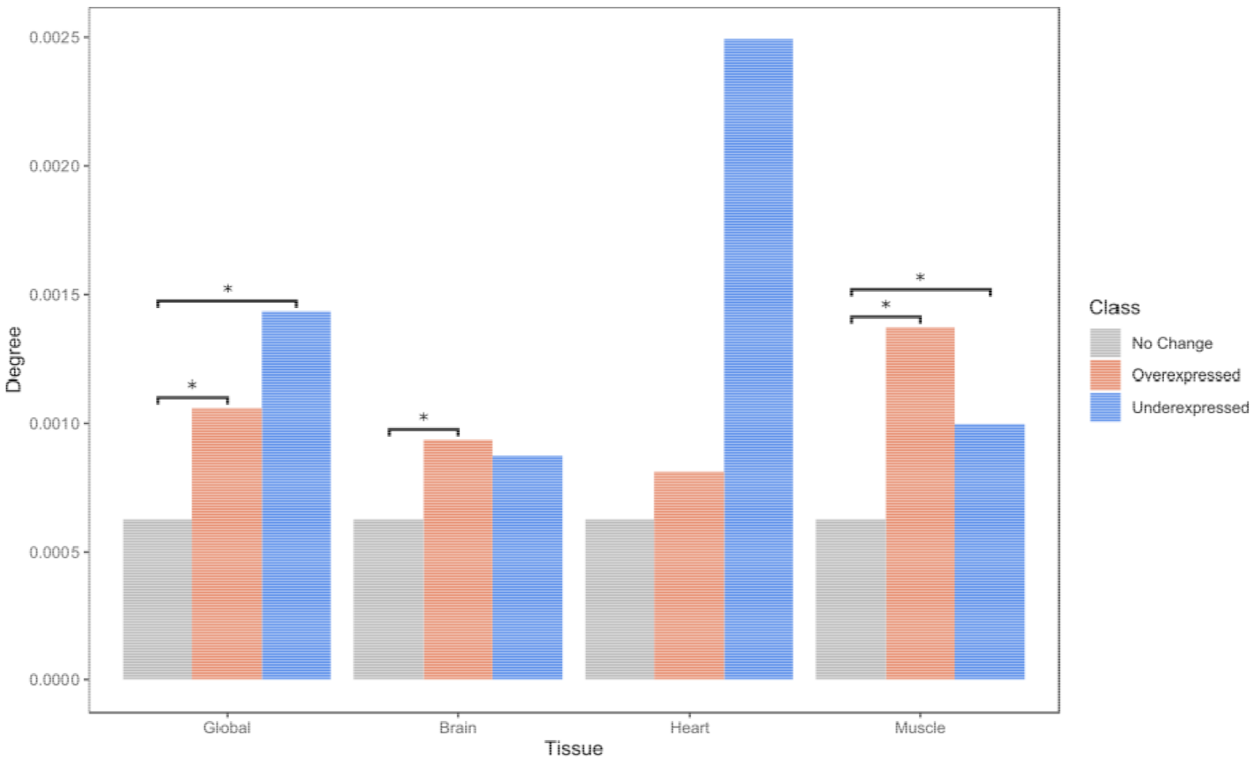

Supplementary Figure 8. Median degree (normalised by dividing by the maximum degree of a graph n-1, where n is the number of nodes in graph G) values in a genome-wide PPI network for overexpressed, underexpressed and unchanged genes from each analysis. * indicates significance tested by a Mann-Whitney U test (Bonferroni corrected).

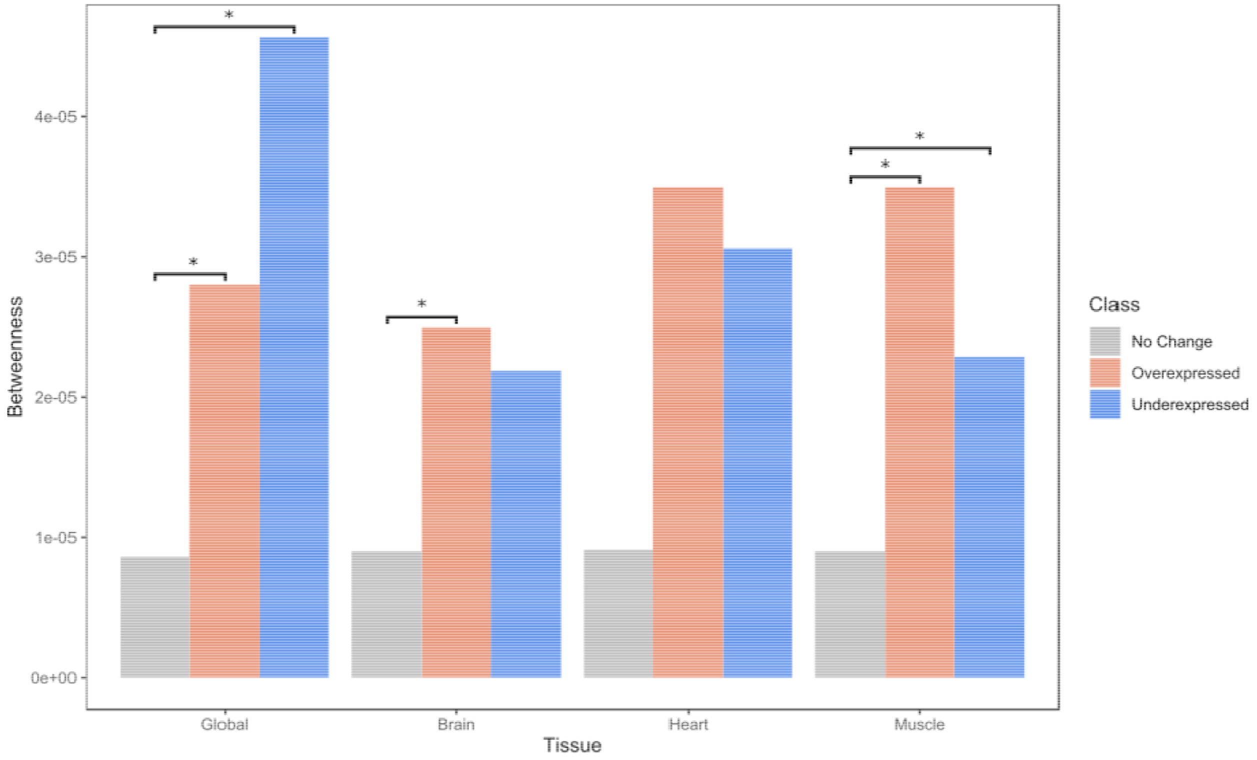

Supplementary Figure 9. Median betweenness values in a genome-wide PPI network for overexpressed, underexpressed and unchanged genes from each analysis. * indicates significance tested by a Mann-Whitney U test (Bonferroni corrected).

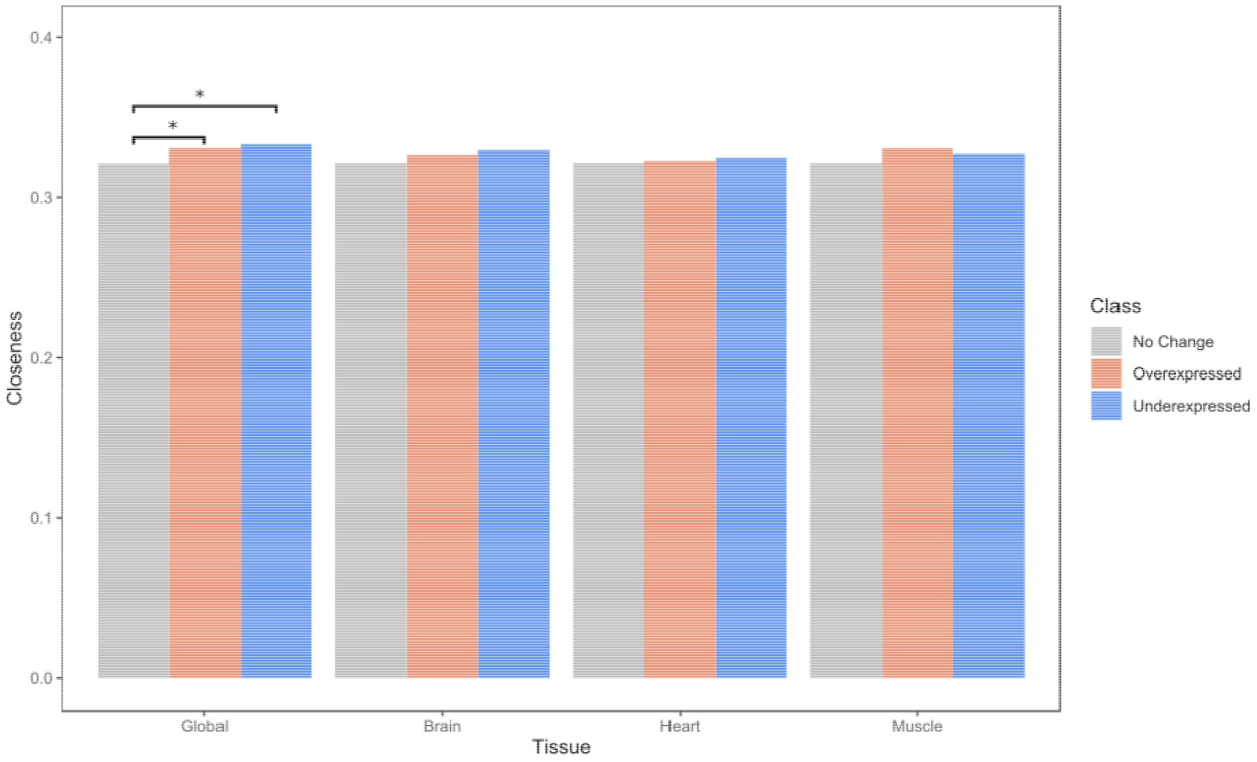

Supplementary Figure 10. Median closeness values in a genome-wide PPI network for overexpressed, underexpressed and unchanged genes from each analysis. * indicates significance tested by a Mann-Whitney U test (Bonferroni corrected).

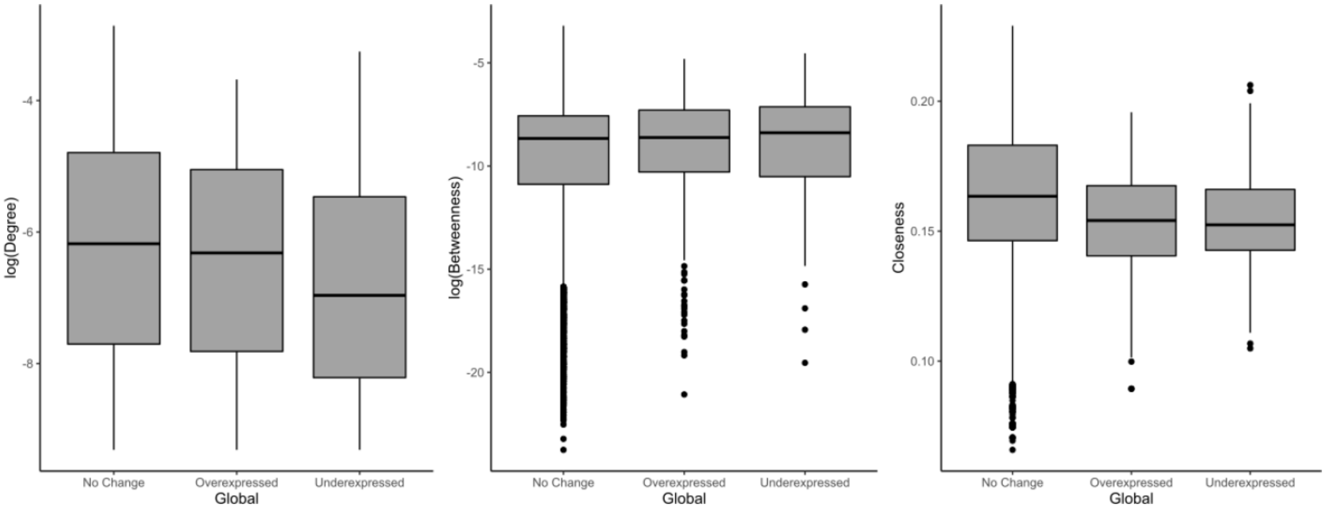

Supplementary Figure 11. Distributions of degree (normalised by dividing by the maximum degree of a graph n-1, where n is the number of nodes in graph G) (**A**), betweenness (**B**) and closeness (**C**) centrality measures in an unweighted co-expression network extracted from GeneFriends for overexpressed, underexpressed and unchanged genes from the global analysis.

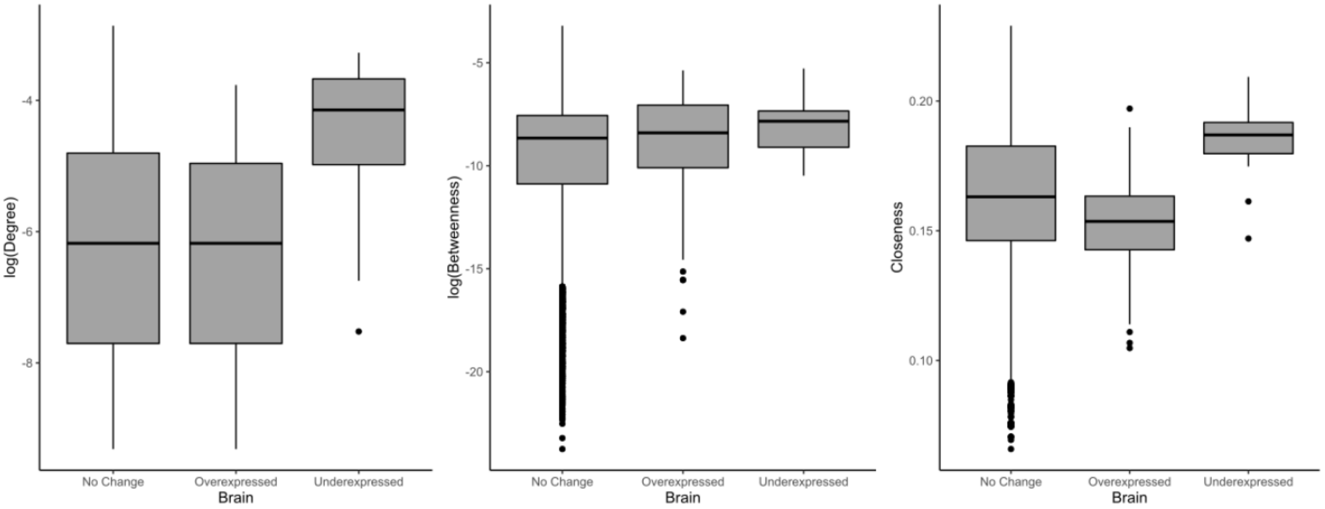

Supplementary Figure 12. Distributions of degree (normalised by dividing by the maximum degree of a graph n-1, where n is the number of nodes in graph G) (**A**), betweenness (**B**) and closeness (**C**) centrality measures in an unweighted co-expression network extracted from GeneFriends for overexpressed, underexpressed and unchanged genes from the brain analysis.

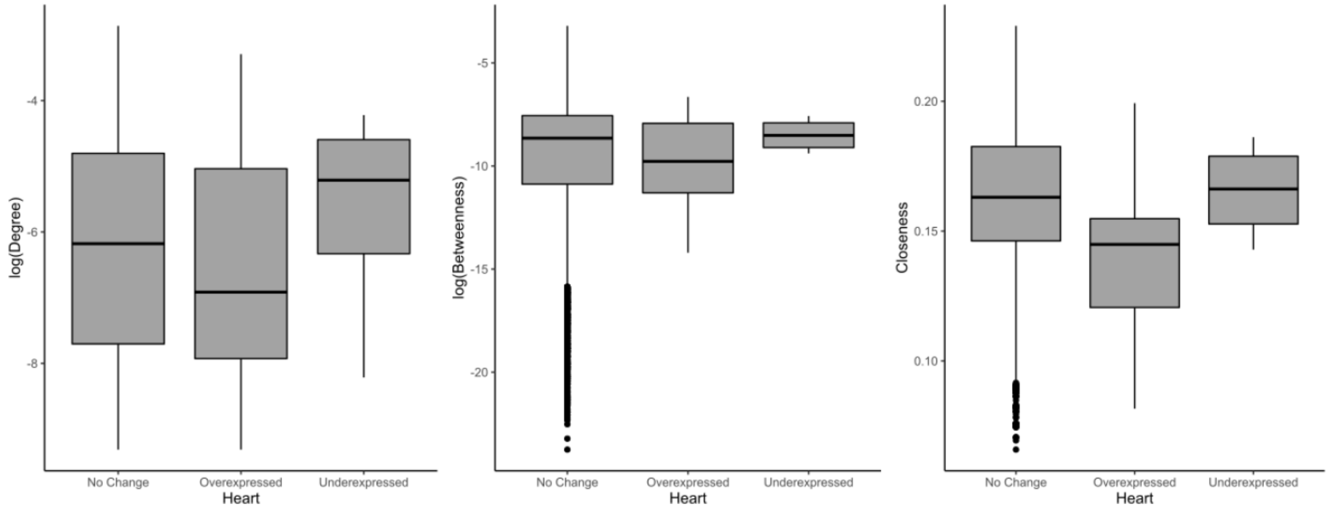

Supplementary Figure 13. Distributions of degree (normalised by dividing by the maximum degree of a graph n-1, where n is the number of nodes in graph G) (**A**), betweenness (**B**) and closeness (**C**) centrality measures in an unweighted co-expression network extracted from GeneFriends for overexpressed, underexpressed and unchanged genes from the heart analysis.

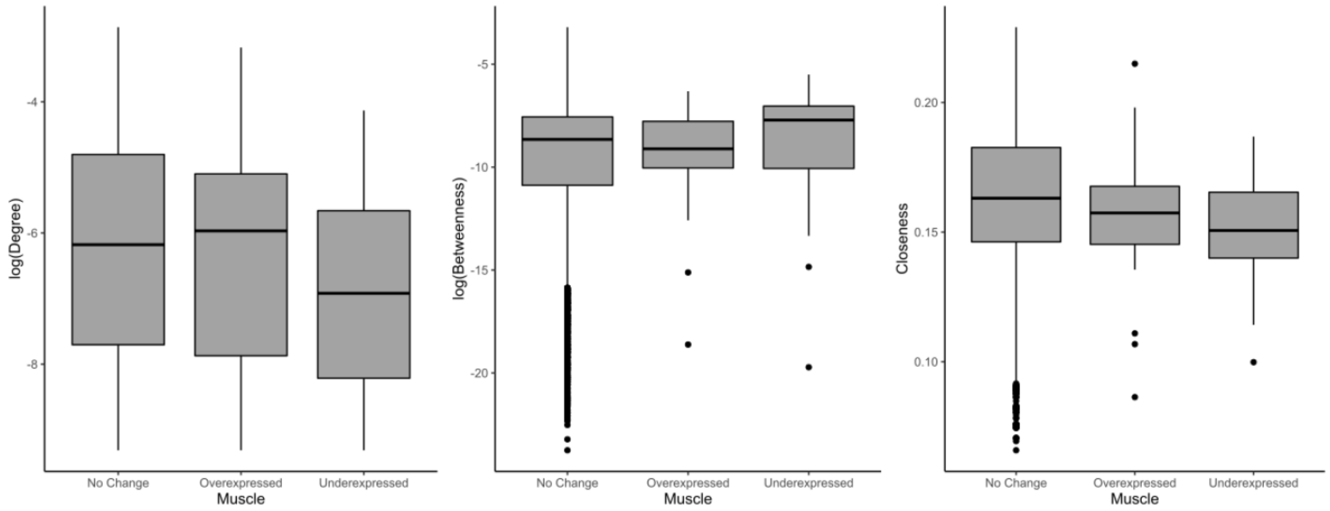

Supplementary Figure 14. Distributions of degree (normalised by dividing by the maximum degree of a graph n-1, where n is the number of nodes in graph G) (**A**), betweenness (**B**) and closeness (**C**) centrality measures in an unweighted co-expression network extracted from GeneFriends for overexpressed, underexpressed and unchanged genes from the muscle analysis.

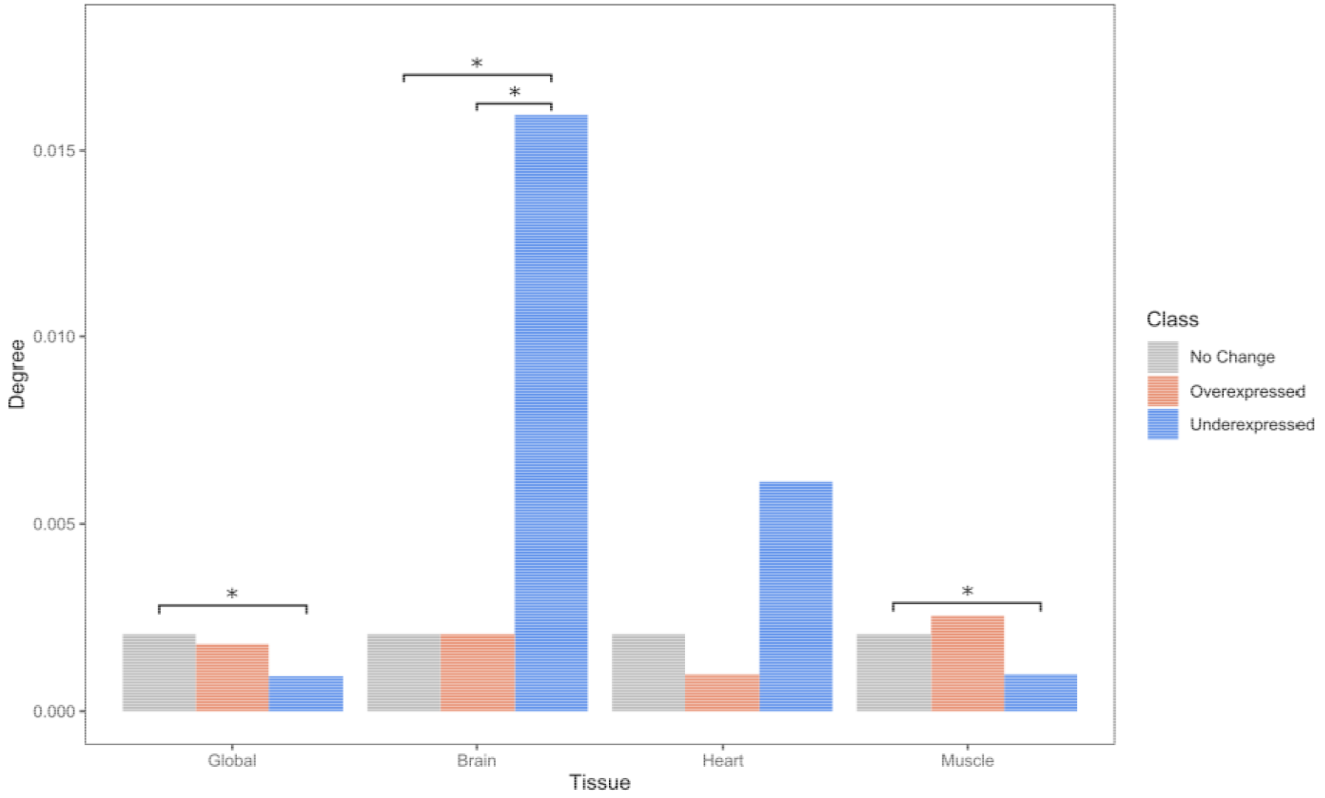

Supplementary Figure 15. Median degree (normalised by dividing by the maximum degree of a graph n-1, where n is the number of nodes in graph G) values in an unweighted co-expression network extracted from GeneFriends for overexpressed, underexpressed and unchanged genes from each analysis. * indicates significance tested by a Mann-Whitney U test (Bonferroni corrected).

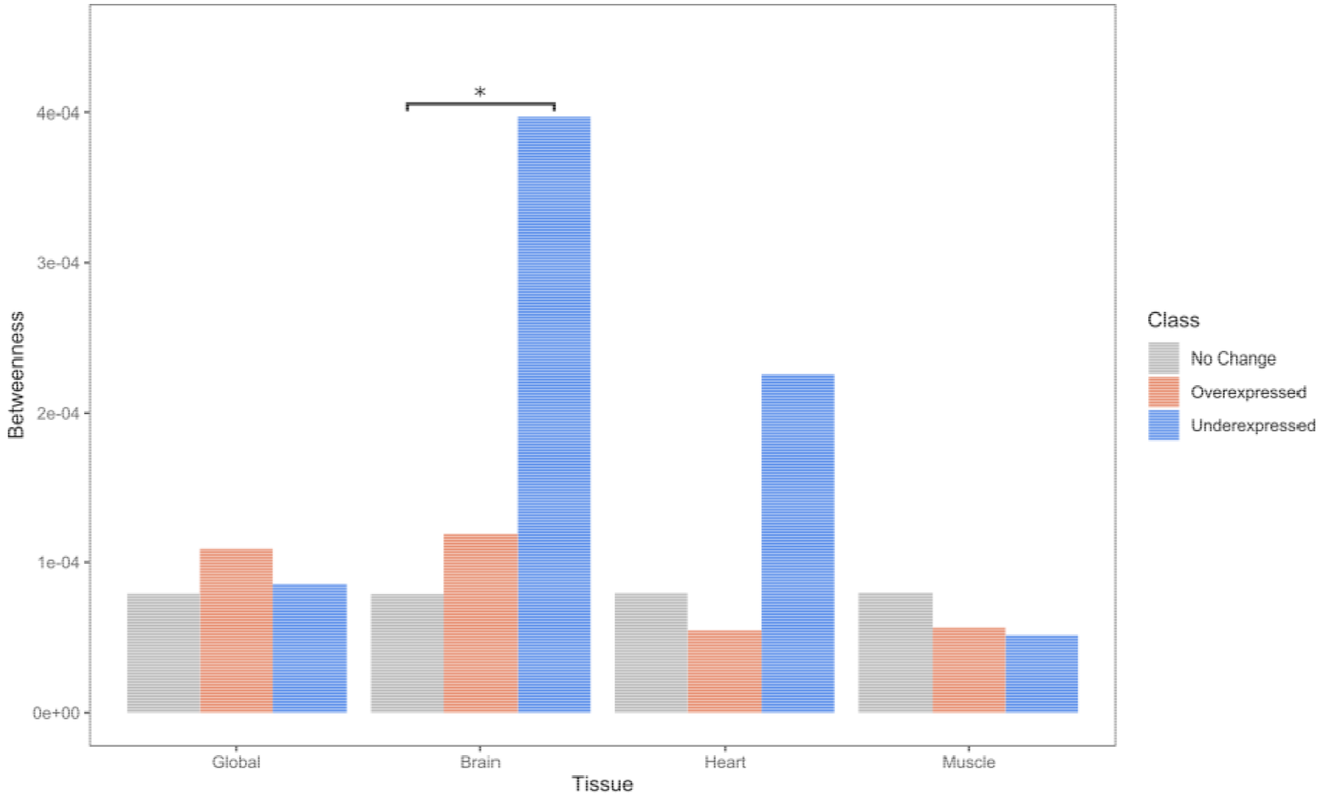

Supplementary Figure 16. Median betweenness values in an unweighted co-expression network extracted from GeneFriends for overexpressed, underexpressed and unchanged genes from each analysis. * indicates significance tested by a Mann-Whitney U test (Bonferroni corrected).

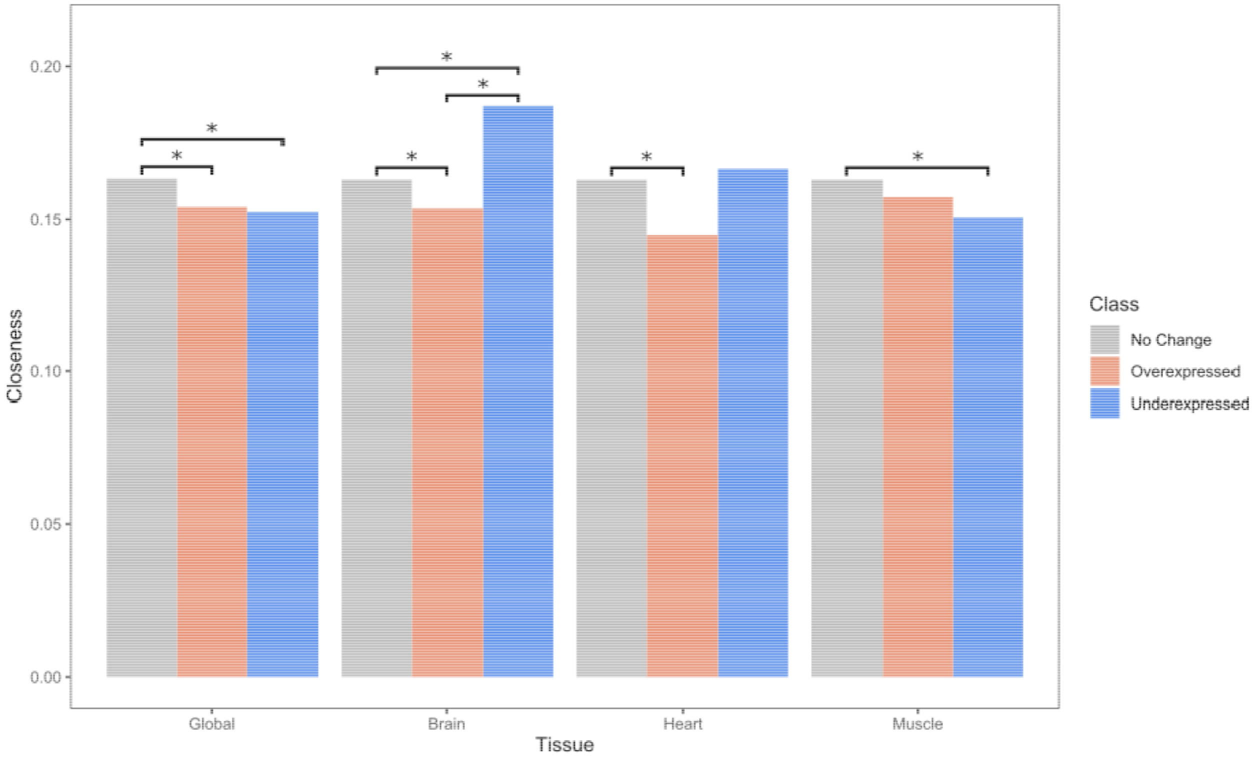

Supplementary Figure 17. Median closeness values in an unweighted co-expression network extracted from GeneFriends for overexpressed, underexpressed and unchanged genes from each analysis. * indicates significance tested by a Mann-Whitney U test (Bonferroni corrected).

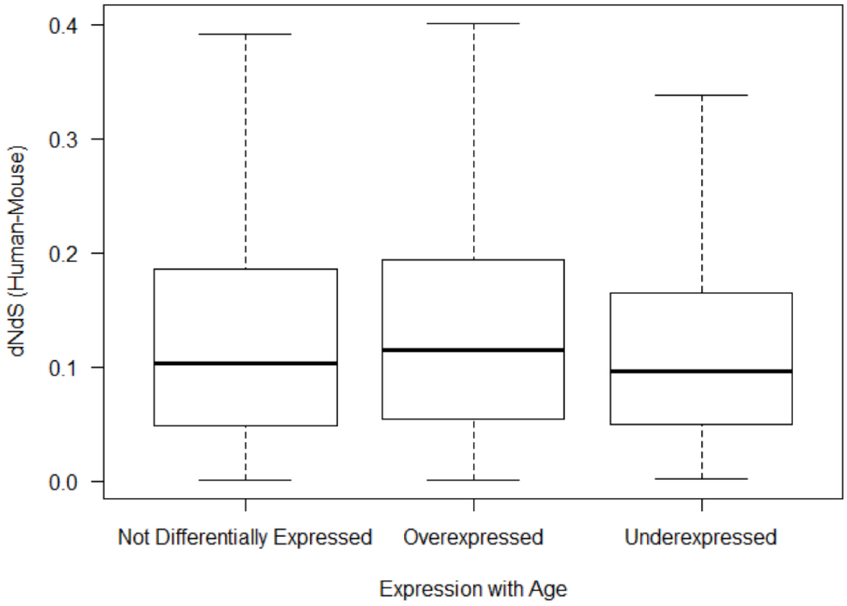

Supplementary Figure 18. Distribution of human-mouse dNdS scores for the different gene classifications (not differentially expressed, overexpressed and underexpressed).

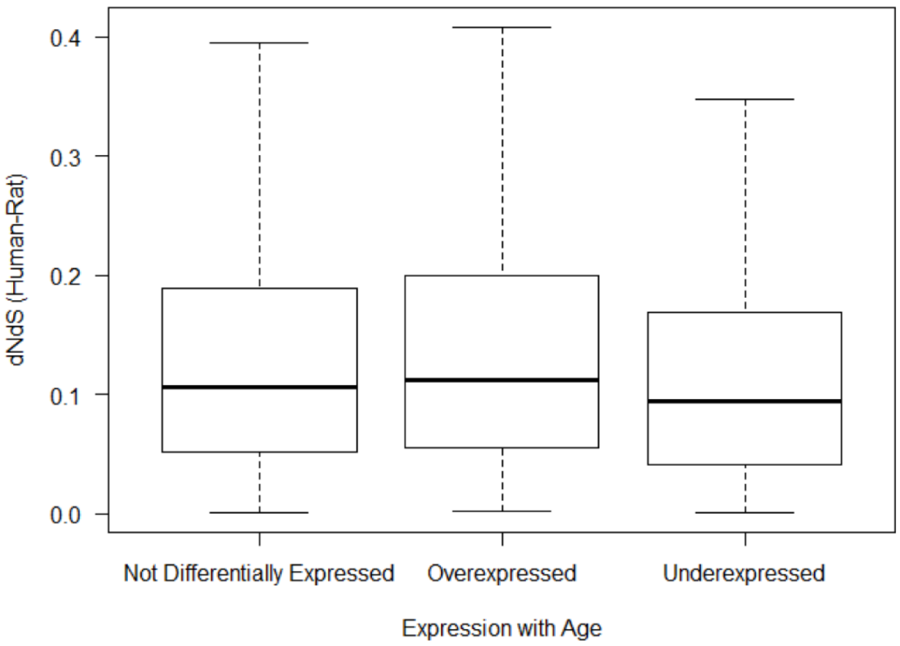

Supplementary Figure 19. Distribution of human-rat dNdS scores for the different gene classifications (not differentially expressed, overexpressed and underexpressed).
